# Healthy weight in pregnancy: time for a shift in approach?

**DOI:** 10.1111/1471-0528.15793

**Published:** 2019-04-29

**Authors:** AP Brown, RM Reynolds, FC Denison

**Affiliations:** ^1^ Tommy's Centre for Maternal and Fetal Health MRC Centre for Reproductive Health Queen's Medical Research Institute Edinburgh UK; ^2^ Simpson Centre for Reproductive Health, Royal Infirmary Edinburgh UK; ^3^ British Heart Foundation Centre for Cardiovascular Science Queen's Medical Research Institute Edinburgh UK

Obesity is one of the biggest challenges facing global reproductive health. Women in the UK and USA are today more likely to be obese or overweight at booking than normal weight, and many low‐ and middle‐income countries (LMICs) seem destined to follow suit (Poston et al. *Lancet Diabetes Endocrinol* 2016;4:1025–36). Understanding how, and to what extent, maternal body mass index (BMI) and weight gain during pregnancy contribute to adverse outcomes for mothers and their offspring is therefore vital to informing future health policy.

In an individual participant data meta‐analysis of over 265 000 births, Santos et al. (*BJOG* 2019; 126:984–95) confirm strong correlations between pre‐pregnancy maternal BMI and the risks of gestational hypertension, pre‐eclampsia and gestational diabetes. Over one‐third of such complications in the study population were considered attributable to maternal overweight and obesity. The risk of large size for gestational age (LGA) at birth increased similarly across all categories of pre‐pregnancy BMI and gestational weight gain, although these data should be interpreted in the context of a continuing debate regarding the customisation of fetal growth charts. It remains uncertain how maternal height and weight influence fetal growth potential, and whether LGA babies born to mothers who are obese or mothers with excessive weight gain carry the same short‐ and long‐term health risks as LGA babies born to mothers who are normal weight. Preterm birth was also more common among women who are obese and past literature has suggested that this association is strongest for extremely preterm delivery (Cnattingius et al. *JAMA* 2013;309:2362–70), whether spontaneous or iatrogenic.

Whereas women who are obese or have high weight gain are consistently shown to be at greatest risk, there is clear evidence of a continuum of risk across the full BMI range, which is emphasised by the authors’ use of population attributable risk (PAR). Notably, the overall burden of pregnancy complications is similar in overweight and obese groups (PAR 11.4 and 12.5%, respectively). This calls into question traditional models of care targeting women with a booking BMI above 30 kg/m^2^ or even higher thresholds. Minimising gestational weight gain in these women ameliorates but does not remove the excess risk, and ultimately may have less impact on outcomes at a population level than previously hoped.

The authors acknowledge that the data were derived from cohorts who were largely white; however, comparable findings have been reported in LMICs with varied ethnic populations (Rahman et al. *Obes Rev* 2015;16:758–70).

Being healthy entails more than just not being obese, and the study also draws important attention to the risks of small size for gestational age and preterm birth, particularly amongst underweight women with inadequate weight gain during pregnancy. These findings strengthen the argument for novel public health approaches to optimise maternal health with a shift in focus towards pre‐conception and interpregnancy interventions delivered in the community, as outlined in a recent review (Hanson et al. *Lancet Diabetes Endocrinol* 2017;5:65–76). Regardless of BMI, all women have the capacity to make their pregnancy healthier and should be empowered to do so through improved access to lifestyle advice and interventions that are tailored to individual needs.

## Disclosure of interests

None declared. Completed disclosure of interests form available to view online as supporting information.
